# Vibrational spectroscopy data fusion for enhanced classification of different milk types

**DOI:** 10.1016/j.heliyon.2024.e36385

**Published:** 2024-08-15

**Authors:** Saeedeh Mohammadi, Aoife Gowen, Colm O'Donnell

**Affiliations:** School of Biosystems and Food Engineering, University College Dublin, Belfield, Dublin 4, Ireland

**Keywords:** NIR, MIR, Raman, PCA, PLS-DA, SO-PLS-LDA, Data fusion

## Abstract

The aim of this study is to classify seven types of Irish milk (butter, fresh, heart active, lactose free, light, protein, and slimline), supplied by a specific company, using vibrational spectroscopy methods: Near infrared (NIR), mid infrared (MIR), and Raman spectroscopy. In this regard, chemometric methods were used, and the impact of spectral data fusion on prediction accuracy was evaluated. A total of 105 samples were tested, with 21 used in the test set. The study assessed principal component analysis (PCA), partial least square discriminant analysis (PLS-DA), and sequential and orthogonalized partial least squares linear discriminant analysis (SO-PLS-LDA) for classifying different milk types. The prediction accuracy, when applying PLS-DA on individual blocks of data and low-level fused data, did not exceed 85.71 %. However, implementing the SO-PLS-LDA strategy significantly improved the accuracy to 95 %, suggesting a promising method for the development of classification models for milk using data fusion strategies.

## Highlights

• Classification of milk types.
• Vibrational spectroscopy on milk.
• Chemometric analysis of vibrational spectroscopic data.
• Fusion of spectroscopic data for classification of different milk type.

## Graphical abstract

**Figure:**
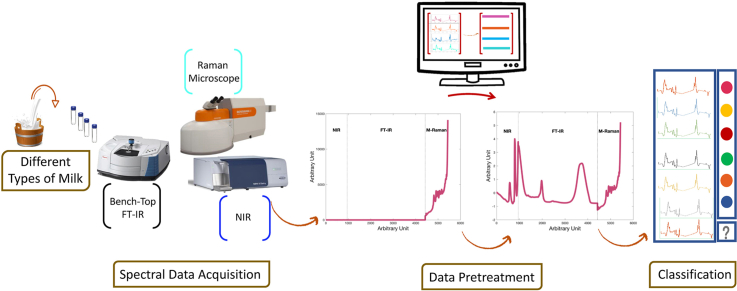

## 1 Introduction

Obtaining qualitative and quantitative information about milk in dairy industries has gained increasing attentions due to the economic benefits of these information [1,2]. In addition, authentication of milk as an increasingly consumed and essential food for humans is of great importance [3]. In this regard, classification of milk samples, whether in liquid or powder form, has drawn significant attention from researchers due to concerns about potential fraud or adulteration [[4], [5], [6]]. Although several analytical methods are available for milk analysis, including gas chromatography [7], liquid chromatography combined with mass spectrometry [8], and nuclear magnetic resonance [9], spectroscopic methods such as NIR, MIR, and Raman spectroscopy offer more rapid and non-destructive alternatives [10,11]. These methods are particularly useful for various purposes, such as measuring composition, authenticating products, detecting adulterants, and assessing degradation through packaging [[12], [13], [14], [15]]. In addition, infrared spectroscopy is a widely used spectroscopic technique in quality assessment laboratories for the authentication of food [16]. A number of classification studies on milk samples using vibrational spectroscopy will be discussed in the following paragraphs.

In a research study, conducted by Chen et al. (2021), 6 different brands of liquid milk in the market have been classified with 100 % prediction accuracy using NIR and chemometrics methods. They applied extreme learning machine (ELM) as well as its ensemble version (EELM) and also PCA for this approach and found it as a promising tool that can be used instead of the time-consuming classic methods [17]. Hosseini et al. (2021) has also exploited NIR for classification of different milk samples adulterated with anionic surfactant. For this purpose, they used machine learning strategies like interval partial least squares (iPLS), PCA, and soft independent modelling of class analogy (SIMCA) to classify 3 groups of samples; one of which belongs to pure milk and the other two groups were adulterated with different levels of the surfactant. The classification accuracy for pure milk were 100 % while mean sensitivity, specificity, and efficiency rate for other groups were 97.6 %, 93 %, and 94.9 %, respectively [18].

Lima et al. (2022) used FT-IR for classification of liquid milk adulterated with cheese whey applying Classification Tree and multilayer perceptron neural networks and their reported results for both methods are precision, sensitivity, and specificity which are above 95 % [19]. Balan et al. (2020) have done a classification study for detecting liquid milk adulterated with formalin, which has a preservative effect, using ATR FT-IR spectroscopy. In this regard, they used SIMCA and PCA and acquired 100 % of classification efficiency [20].

In addition to adulteration, differentiation between milk of various animals have been subject of spectroscopic studies. In a research study FT-IR has been employed for discrimination between mixtures of goat-cow and buffalo-cow milk and gained classification rate of 93 % and 91 %, respectively, using orthogonal partial least square discriminant analysis (OPLS-DA) [21]. Raman spectroscopy have also been studied by Amjad et al. for classification of liquid milk belongs to various species including cow, goat, buffalo, and human. In this regard, the average accuracy, precision, specificity, and sensitivity have been obtained as about 93.7 %, 94 %, 97 % and 93 %, respectively [22]. Yazgan et al. (2020) utilized Raman spectroscopy in combination with PLS-DA to differentiate between pasteurized and raw milk, as well as to distinguish milk from different species including cow, goat, ewe, and their mixtures. They achieved a prediction accuracy higher than 91.5 %, demonstrating the potential of Raman spectroscopy and PLS-DA in classifying these samples [23].

Data fusion strategies have been also used for classification of different foods and beverages in different studies [[24], [25], [26], [27], [28]]. In this regard, combining data from different sources, each providing complementary information about the samples, will offer more insights into the samples and lead to more robust models [29]. In one study classification of milk obtained from cows fed with different diet has been done using fusion of data acquired from two modes of mass spectrometry [26].

Although there have been several studies for classification of liquid milk for both adulteration detection and species discrimination, there is not any study for classification of different liquid milk types for finished products of the same brand which can be used for authentication of the wide range of liquid milk products offered by dairy companies. Moreover, fusion of spectroscopic data for classification of liquid milk has not been assessed yet. In this study, classification of seven types of Irish milk from the same brand have been evaluated by applying vibrational spectroscopy including NIR, FT-IR and Raman. For this purpose, chemometric methods such as PCA, PLD-DA, and SO-PLS-LDA have been investigated.

## 2 Materials and methods

### 2.1 Experimental data acquisition

In this research, the classification of seven types of commercial liquid milk by using three different spectroscopic instruments and applying chemometric methods have been studied. In this regard, 7 types of liquid milk including butter, fresh, heart active, lactose-free, light, protein and slim line prepared from the market and were analyzed using, a Raman microscope (Renishaw inVia), near-IR (Bruker, MPA II), and bench-top FT-IR systems (Thermo Fisher is50). The samples were acquired one day prior to the analysis and stored in a refrigerator set at 3 °C. They were portioned into plastic tubes and remained in the refrigerator until the time of analysis. For the NIR analysis, samples were sonicated in a water bath set at 40 °C for 20 min. The NIR and FT-IR spectra were recorded in wavenumber range of 12,000 to 3800 cm^−1^ and 400 to 4000 cm-1, respectively. The emitting laser wavelength used for Raman spectroscopy was 785 nm, and the Raman spectra obtained in the wavenumber range of 190–2412 cm^−1^, over the acquisition time of 20 s. The experimental set-up was optimized separately for each instrument and five independent measurements have been taken using five sets of distinct samples.

There were seven distinct types of milk, each from a different product batch. For each type of milk, in 5 separate days, only one packet was used. Over the course of five separate days, three different samples were taken from the same packet of each milk type. Each of these samples was then analyzed independently. Therefore, for each day, each type of milk has been undergone three independent measurements. Over the five days, each individual sample was measured a total of fifteen times. Therefore, there were fifteen spectra for each sample as the whole experiment were repeated five times and through each repetition, three independent measurements have been carried out for each sample. The first and second measurements were conducted in June, the third measurement in October, and the fourth and fifth measurements in November 2022.

**Fig. 1:**
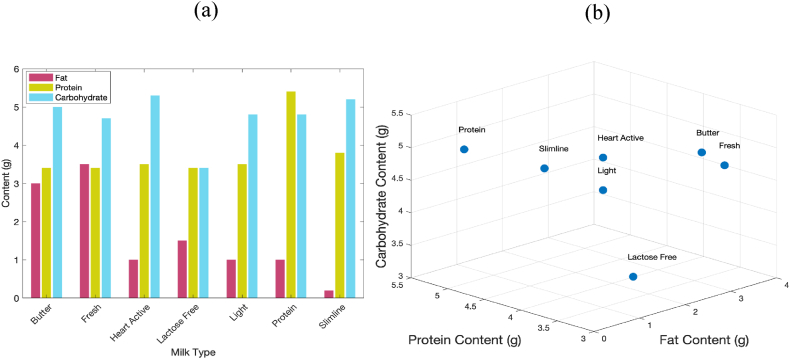
(a)Bar plot of fat, protein, and carbohydrate for 7 types of milk (b) 3D scatter plot of different types of milk based on the values of carbohydrate, protein, and fat content.

Table S1 shows the nutrition contents of 7 different types of milk as claimed on their nutrition fact labels. In addition, the bar plot, and 3D scatter plot of different types of milk based on three principal ingredients of all milk types, have been shown in Fig. 1a and b. As can be observed in this Figure, lactose free milk is distinguishable from other types of milk considering the main ingredients.

### 2.2 Data analysis

NIR, MIR, and Raman spectroscopic data were analyzed both individually and in combination to assess the effectiveness of data fusion strategies. For the data analysis, spectra from each modality, obtained from four measurements, were augmented column-wise to form a single matrix for the training set, while the spectra from one measurement were reserved as the test set. The third measurement was selected as the test set for data analysis. To combine the spectra, each block of augmented spectra was concatenated row-wise. Data analysis methods such as PCA, PLS-DA, and SO-PLS-LDA were utilized. All spectra underwent standard normal variate (SNV) pretreatment before PCA analysis. Additionally, for PLS-DA analysis, various pretreatments, including SNV, first and second derivatives (1D and 2D) of Savitzky–Golay, SNV following first and second derivatives of Savitzky–Golay, and multiplicative scatter correction (MSC), were applied to evaluate their impact on result accuracy. For SO-PLS-DA, cross-validation was conducted using a systematic approach, specifically the Venetian blind method, which involved dividing the dataset into multiple segments. Each segment was used sequentially as the validation set, with the remaining parts of the data serving as the training set. This method ensures that every part of the dataset is used for both training and validation, enhancing the reliability of the model evaluation by minimizing bias and variance in the assessment of model performance. Optimal model complexity, crucial for ensuring adequate model performance without overfitting, was determined based on the classification error observed during the cross-validation process. The selection of the number of latent variables, which are integral to the model's structure, was driven by the objective of minimizing the classification error across the validation segments. This approach balances the model's ability to generalize to new data while retaining sufficient complexity to capture essential patterns and relationships in the training dataset.

In the SO-PLS-LDA approach, auto scaling and mean centering were employed to assess the influence of these pretreatment methods on prediction accuracy. The optimum number of latent variables in different blocks varies, and they are sequentially selected based on cross-validation. In SO-PLS, the first block is modeled using PLS, after which the subsequent block is orthogonalized based on the scores of the previous block. In the PLS-LDA part, linear discriminant analysis is performed on the score plot obtained from PLS analysis. This approach leads to the extraction of stable and relevant components for classification and helps overcome the problem of dimensionality [30,31].

All data analysis procedures were performed using MATLAB software R2022a and R2023a. For the SO-PLS-DA analysis, the Sonic High-Performance Computing (HPC) cluster at University College Dublin (UCD) was also utilized. The SO-PLS-DA MATLAB scripts were obtained from the https://www.chem.uniroma1.it/romechemometrics/website.

## 3 Results and discussion

### 3.1 Visual representation of raw, pretreated, and fused spectra

**Fig. 2:**
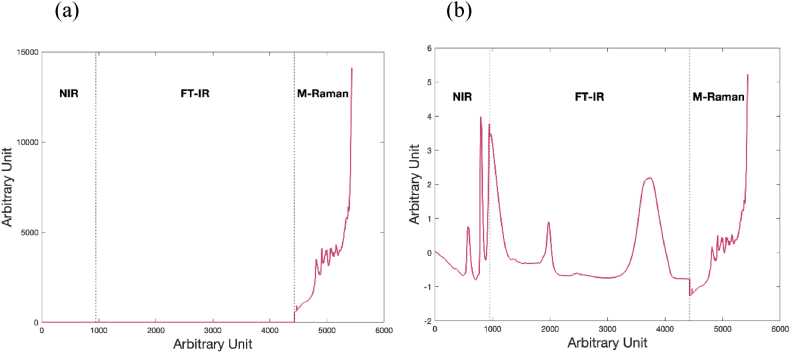
The spectra of fused NIR, FT-IR, and Raman data. Part (a) shows the raw data, and part (b) displays the data after SNV pretreatment.

**Fig. 3:**
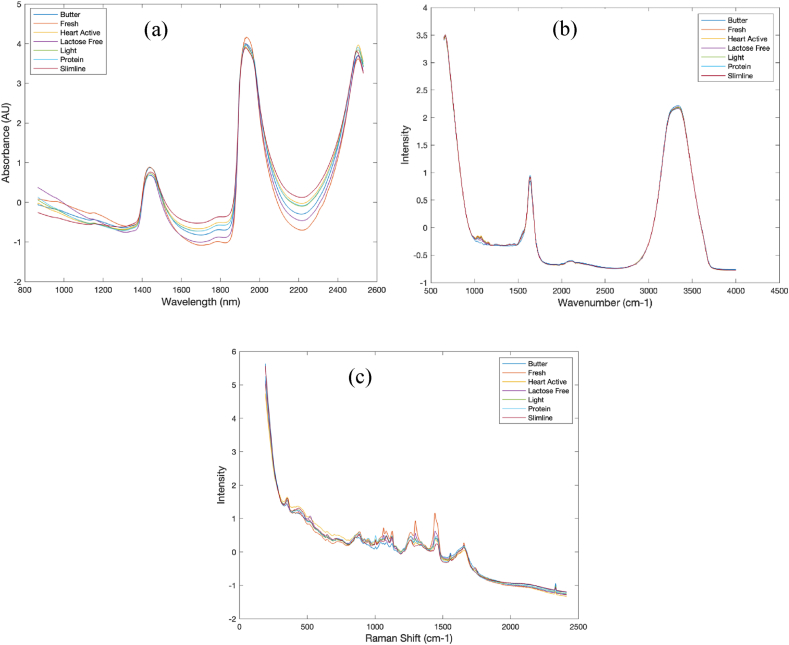
The mean (a) NIR, (b) MIR, and (c) Raman spectra from five experiments for each group after SNV pretreatment.

The raw NIR, FT-IR, and Raman spectra for all types of samples are depicted in Table S2. These spectra are the average of fifteen independent measurement of each type of samples. Spectra obtained from each technique have their own specific scales, and, thus, they should be scaled using a proper pretreatment method before data fusion [32]. Fig. 3a represents the spectra of fused data without a specified unit for the x-axis. As can be seen in this Figure the scale difference between different spectra does not allow to observe details of the NIR and FT-IR spectra. This suggests that the fused spectra might benefit from adjustments in scale to enable a more detailed analysis of the spectra. In this regard, the effect of SNV pretreatment on the fused spectra can be observed in Fig. 2b.

Fig. 3a to 3 c presents the mean NIR, MIR, and Raman spectra from five experiments for each group after SNV pretreatment. In NIR spectroscopy, peak assignments for milk composition analysis cover a range of functional groups and bond vibrations related to key milk constituents. The peaks at 1490 nm and 2100 nm, representing the first overtone and combination bands of OH stretching vibrations, typically indicate the presence of lactose, a major sugar in milk. Similarly, protein content in milk is revealed through peaks associated with amide groups, such as the Amide B and Amide I bands at 1640–1670 nm and 2056 nm, which represent the first overtone of NH stretching and NH combination vibrations. Fats are identifiable through CH stretching vibrations at 1660 and 1730 nm, indicating the molecular vibrations of fatty acids. These NIR spectra are crucial for quickly assessing milk's primary macronutrients-proteins, fats, and carbohydrates-offering a non-destructive and rapid analysis tool [33].

Milk consists of approximately 88 % water, which produces notably strong bands in the NIR spectrum at wavelengths of 960, 1440, 1950, and 2076 nm. These bands can overlap with other significant bands, introducing noise into the measurements [[33], [34], [35]]. In the MIR spectrum, the first water band overlaps with the relatively smaller amide I and amide II protein bands, which are located in the 5882–6250 nm and 6369–6451 nm ranges, respectively [33,36]. Furthermore, the absorption of infrared light by target analytes in milk is influenced by the concentration and size of fat globules, which contribute to light scattering and account for up to 50 % of the total absorbance in NIR at wavelengths of 1454, 1894, 1953, 2048, 2100, 2174, and 2230 nm [33,37,38]. These interferences can impact the precision and/or accuracy of the analytical outcomes.

In MIR spectroscopy, the analysis becomes more detailed with broader peak assignments due to the fundamental vibrations of molecular bonds. For example, the Fat-B peak at 3500 cm^−1^ and Fat-A peak at 5700 cm^−1^ are significant for the stretching vibrations of saturated C–H bonds and C=O ester groups in fatty acids, respectively. Protein analysis in MIR uses Amide I, II, and III bands located at 6060 cm-1, 6500 cm^−1^, and 8064 cm^−1^, which provide insights into the protein structure through C=O stretching, N–H bending, and C–N stretching vibrations. Carbohydrates show peaks such as the C–O–C ether stretching at 8000 cm^−1^ and 8643 cm^−1^, important for understanding the structure and interaction of lactose molecules. MIR spectroscopy, with its detailed vibrational analysis of molecular bonds, provides a comprehensive chemical profiling tool for milk, crucial for quality control and nutritional analysis [33].

The direct interpretation of spectra is challenging due to the complex nature of the milk matrix. Nonetheless, the spectra obtained in a study demonstrate a consistent and clear downward trend associated with lactose levels [15]. Specifically, for all samples examined, variations in lactose were noticeable in peaks below 700 cm^−1^, which correspond to skeletal signals and both endocyclic and exocyclic deformation bands [39]. Additionally, around 1100 cm^−1^, there are vibrational modes of carbohydrates influenced by C–C and C–O bonds [[40], [41], [42], [43]], and between 1500 and 1300 cm^−1^, there are deformation vibrations of HCH and CH2OH groups [40,42]. Moreover, similar spectral regions were identified by Vaskova and Buckova (2016) in their comparison of dried whole milk and lactose-free milk. They noted that the range from 400 to 600 cm^−1^ corresponds to endocyclic and exocyclic deformations, while the signals at 918 cm^−1^ and approximately 1070–1090 cm^−1^ relate to glucose, with a particularly strong signal at 1087 cm^−1^ attributed to C–O–H bending mode vibrations in lactose [15,44].

### 3.2 Principal component analysis (PCA)

**Fig. 4:**
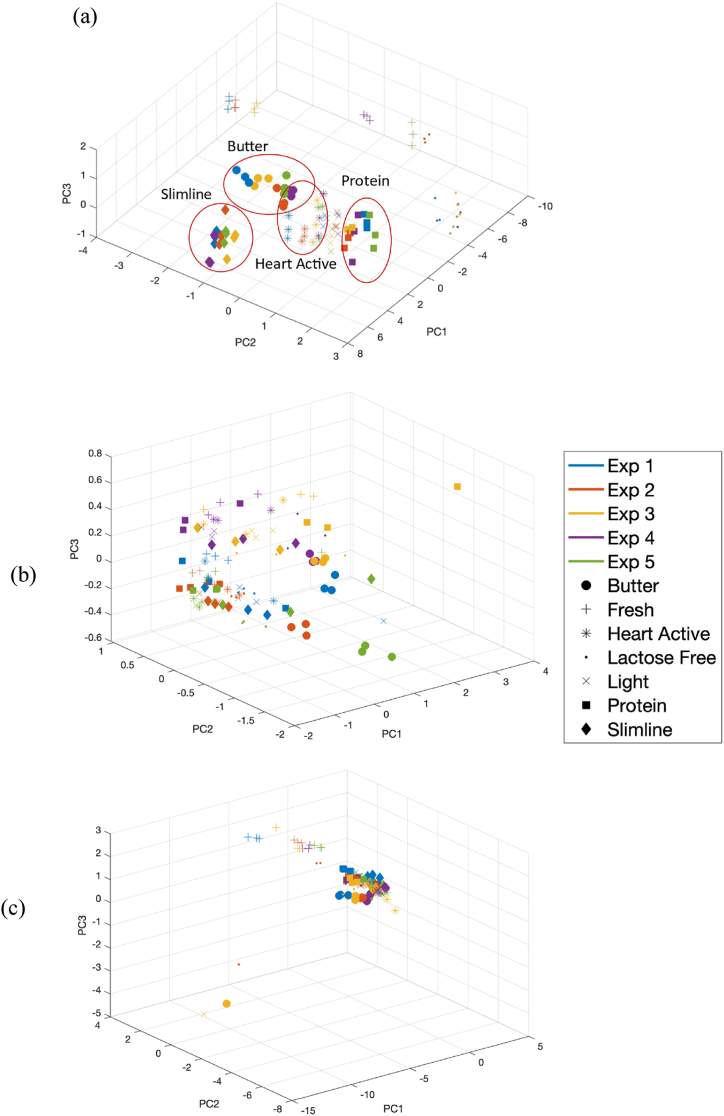
The score plot of PC1 versus PC2 versus PC3 for (a) NIR, (b) FT-IR, and Raman spectra after SNV. Repetition from 1 to 5 are depicted with different colors. Butter, fresh, heart active, lactose free, light, and protein milk have been shown with circle, plus, star, point, cross, square, and diamond, respectively.

The score plot of PC1 versus PC2 versus PC3 are shown in Fig. 4 a to c for NIR, FT-IR, and Raman spectroscopy, respectively. As can be seen in this Figure the most visual differentiation between different types of milk is associated with NIR data (Fig. 4, a). In this regard, butter, heart active, lactose free, and protein milk have been totally separated from other groups using NIR and PCA.

**Fig. 5:**
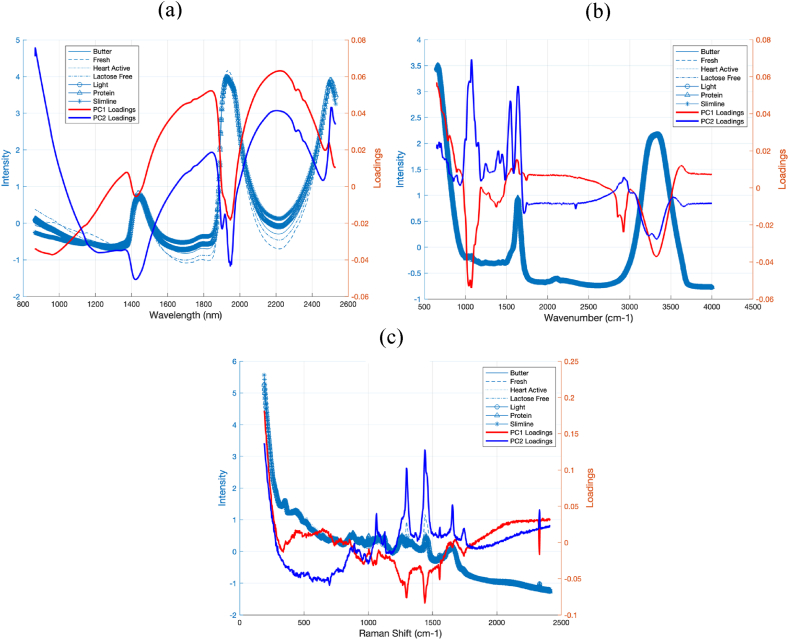
The loadings values corresponding to principal component 1 and 2 obtained from PCA analysis and the mean NIR, MIR, and Raman spectra from five experiments for each group after SNV pretreatment for (a) NIR, (b) FT-IR, and (c) Raman.

The loading plots in Fig. 5 identify the most influential wavenumbers for principal components 1 and 2. Specifically, Fig. 5a shows that the most important wavelengths for PC1 on NIR data, which cause the most variation in the data, are around 1974, 1373, 1425, 1846, and 2212 nm. These wavelengths are within the water region of the spectra. This may be due to variations in water content among different types of milk examined in this study which in turn would cause significant differences in their spectra. Other important wavelengths with high loadings on PC1 are 1820, 2180, and 2320–2350 nm, which are related to lactose, protein, and fat, respectively. For PC2, the pattern of the loading values is similar to that of PC1. The significant difference is that PC2 considers wavelength 869 nm, at the very beginning of the spectra, as an important wavelength [[33], [34], [35], [36], [37], [38]]. For FT-IR data, an important wavenumber for both PC1 and PC2 is around 1076 cm^−1^, probably related to lactose. Therefore, the most variation in FT-IR spectra is due to lactose or carbohydrate. Another important wavenumber for PC1 is 1754 cm^−1^, related to fat, while for PC2, the wavenumbers 1538 and 1650 cm^−1^ are important, related to protein [33]. The important Raman shifts from the loading plot (Fig. 5c) for PC1 are 1062, 1296, 1441, and 1654, and for PC2 are 1298, 1439, and 1555 [15].

**Fig. 6:**
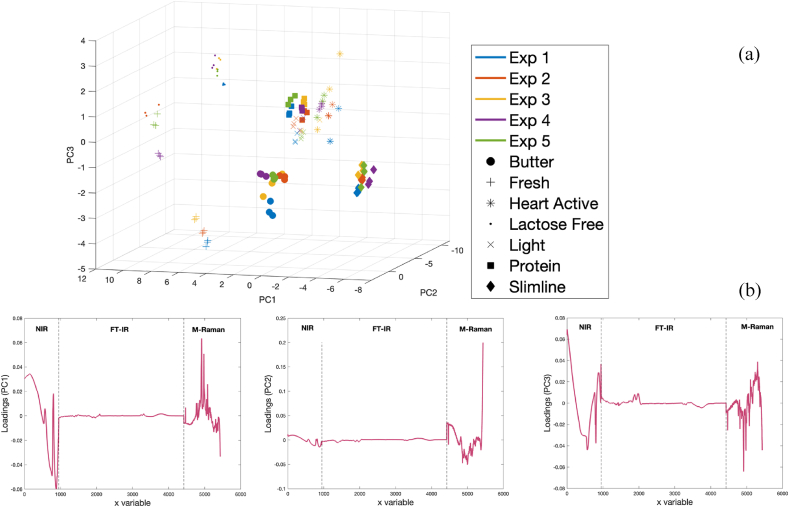
The(a) score and (b) loading plot of PC 1 to 3 obtained from PCA analysis of fused NIR, FT-IR, and Raman spectra after SNV. Repetition from 1 to 5 are depicted with different colors. Butter, fresh, heart active, lactose free, light, and protein milk have been shown with circle, plus, star, point, cross, square, and diamond, respectively.

PCA analysis has been also done on the fused data. In this regard, first SNV pretreatment was used for individual blocks of data, and then, pretreated blocks were fused. Fig. 6a, which is the score plot of PC1 versus PC2 versus PC3 for fused data, reveals that the three data points related to third measurement, shown in blue, is relatively separated from others, making it a proper option for the test set. It is worth noting that the third measurement have been also performed on different month of all other measurements (Table 1). Interestingly, the visual differentiation between different sample types through score plot of fused data seems to be at the same level as NIR data. Fig. 6b shows the loading plot of PC1 to PC3, obtained from PCA analysis of fused NIR, FT-IR, and Raman spectra after SNV normalization. The plot indicates that the first and last parts of the loading plots have higher values, suggesting that NIR and Raman data contribute more to the classification which can be related to higher sensitivity of Raman and NIR techniques to scattering of light in different types of milk in response to variation in compositional properties of samples.

**Table 1:** A summary of the classification models developed and their accuracy of prediction.

Chemometric Method	Spectroscopic Method	Accuracy of Prediction for Test Set (%)	Classes with 100 % Prediction Accuracy
PLS-DA	NIR	71.42	Butter, Fresh, Lactose Free
PLS-DA	FT-IR	71.43	Butter, Lactose Free, Protein
PLS-DA	Raman	85.71	Fresh, Protein, Slimline
PLS-DA	Fused NIR, FT-IR, and Raman	85.71	Fresh, Protein, Slimline
SO-PLS-LDA	Fused NIR, FT-IR, and Raman	95	Fresh, Heart Active, Lactose Free, Protein, Slimline

### 3.3 Partial least squares- discriminant analysis (PLS-DA)

#### 3.3.1 NIR

The NIR data were analyzed using Partial Least Squares Discriminant Analysis (PLS-DA), with the third repetition set serving as the test set. In this regard, the maximum mean class accuracy of the cross validation was obtained 71.75 with 8 latent variables and without any pretreatment (Fig. S1). Considering 8 latent variables the accuracy of the calibration and test set was 79.76 and 71.42. The confusion matrix of the test set with 8 latent variables has been depicted in Fig. S2. All butter, fresh, lactose free, and Slimline milk samples in the test set were predicted correctly with PLS-DA analysis of the NIR data. However, there were two false positive samples in Slimline class which were related to two protein samples predicted as Slimline milk.

#### 3.3.2 FT-IR

The FT-IR data were analyzed using Partial Least Squares Discriminant Analysis (PLS-DA), with the third repetition set serving as the test set. In this regard, the maximum mean class accuracy of the cross validation was obtained 73.78 with 13 latent variables and MSC pretreatment (Fig. S3). With 13 latent variables and MSC pretreatment the accuracy of the calibration and test set was 84.52 and 71.43, respectively. The confusion matrix of the test set has been depicted in Fig. S4. All butter, lactose free, protein, and Slimline milk samples in the test set were predicted correctly with PLS-DA analysis of the FT-IR data. However, there were five false positive samples in Slimline class which were related to one fresh milk sample, three heart active samples and one light milk sample predicted as Slimline milk.

#### 3.3.3 Raman

The Raman data were analyzed using Partial Least Squares Discriminant Analysis (PLS-DA), with the third repetition set serving as the test set. In this regard, the maximum mean class accuracy of the cross validation was obtained 95.38 with 11 latent variables and without any pretreatment (Fig. S5). With 11 latent variables and without pretreatment the accuracy of the calibration and test set was 98.81 and 85.71, respectively. The confusion matrix of the test set has been depicted in Fig. S6. All fresh, lactose free, protein, and Slimline milk samples in the test set were predicted correctly with PLS-DA analysis of the FT-IR data. However, there were two false positive samples in lactose free class which were related to one butter milk sample, and one light milk sample predicted as lactose free milk.

#### 3.3.4 Fused data analysis

**Fig. 7:**
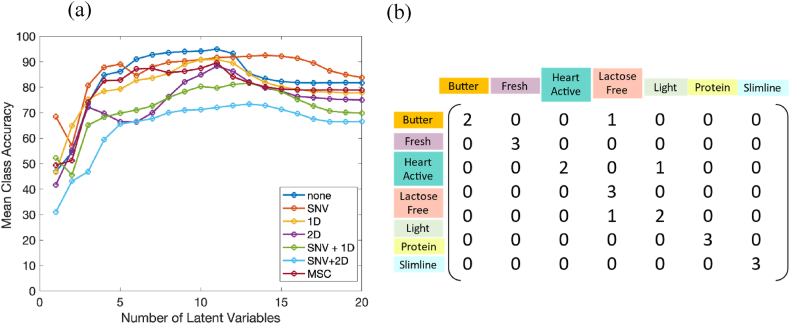
(a). Mean class accuracy versus number of latent variables acquired from PLS-DA analysis of Fused data with cross validation. Each plot corresponds to one of the following: no pretreatment, SNV, first derivative (1D), second derivative (2D), first derivative after SNV, second derivative after SNV, and MSC. (b). The confusion matrix of test set for PLS-DA analysis of Fused Data.

The low-level fused data were analyzed using Partial Least Squares Discriminant Analysis (PLS-DA), with the third repetition set serving as the test set. In this regard, the maximum mean class accuracy of the cross validation was obtained 94.16 with 10 latent variables and without any pretreatment (Fig. 7a). With 10 latent variables and without pretreatment the accuracy of the calibration and test set was 98.81 and 85.71, respectively. The confusion matrix of the test set has been depicted in Fig. 7b. All fresh, lactose free, protein, and Slimline milk samples in the test set were predicted correctly with PLS-DA analysis of the FT-IR data. However, there were five false positive samples in lactose free class which were related to one butter milk sample and one light milk sample predicted as lactose free milk. According to the obtained results, the accuracy of prediction using Raman spectroscopy is comparable with fused NIR, FT-IR, and Raman data. Therefore, in this case study low-level data fusion has not improved the prediction accuracy.

### 3.4 Sequential and orthogonalized- partial least squares- linear discriminant analysis (SO-PLS-LDA)

In this chapter, we analyze datasets that have undergone various forms of preprocessing - no preprocessing, mean cantering, and auto-scaling, which we refer to as none, mean, and auto, respectively. Considering these three preprocessing methods across three blocks of data, a total of 27 unique combinations will be evaluated and discussed.

The data of the third measurement, which was determined as the most different one by PCA, was used as test set. Here, the highest accuracy, which was 0.95, was obtained with three specific combinations for the NIR, FT-IR, and Raman blocks: none, auto, none; none, auto, mean; and mean, mean, none. The optimum count of latent variables across different spectroscopic techniques and various pre-treatment approaches was determined using cross-validation. By assessing the prediction accuracy, specifically through metrics such as RMSECV, the optimal latent variable combination that offers the best predictive performance is identified. This optimal combination is then used to construct the final SO-PLS-LDA model on the entire dataset. For NIR, FT-IR, and Raman blocks with no pre-treatment, the numbers of latent variables were 10, 1, and 6, respectively. This configuration remained unchanged when auto pre-treatment was applied to the FT-IR block and mean pre-treatment to the Raman block. However, when a mean pre-treatment was employed on both the NIR and FT-IR blocks while leaving the Raman block without any pre-treatment, the optimum latent variable counts shifted to 5, 9, and 5, respectively.

**Fig. 8:**
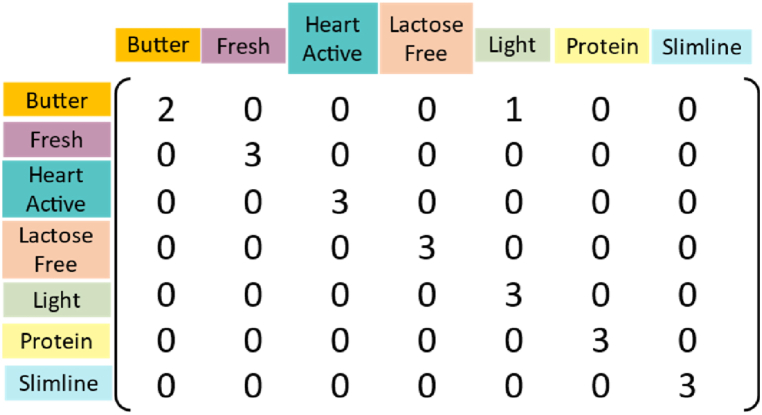
Confusion matrix for classification of different types of milk using SO-PLS-LDA on fused NIR, FT-IR, and Raman spectra with none, auto, and none pretreatment, respectively.

Confusion matrix associated with third group as test set for the combination seven has been displayed in Fig. 8. There is only one butter sample misclassified as light milk. Table S3 reports the obtained accuracy for twenty-seven combinations. SO-PLS-LDA in this case study, by prediction accuracy of 95 % for test set, can be considered as a potential data fusion strategy. A summary of the developed classification methods and their performance in prediction is summarized in Table 1. Although the performance of the prediction has been improved using SO-PLS-LDA, only NIR and FT-IR spectroscopy alone could classify butter milk with 100 % accuracy.

## 4 Conclusion and future outlook

The importance of milk analysis for quality, quantity, and authenticity is growing, given its economic value and essential role in nutrition [1]. In this regard, the potential of spectroscopic techniques, including NIR, FT-IR, and Raman, for classifying various types of Irish milk was evaluated in this study. To achieve precise predictions, different chemometric approaches were applied. In this regard, PCA analysis was used to explore the data and around half of the classes were visually separated by PCA analysis on NIR data. A prediction accuracy of 85.71 % was obtained when PLS-DA was applied to individual blocks of data and low-level fused data. However, implementing SO-PLS-LDA significantly improved the accuracy, suggesting a promising way to develop data fusion methods for milk classification. In this regard, fresh, heart active, lactose free, protein, and slimline milk were classified without any false positive or false negative predictions. The results suggest that SO-PLS-LDA can improve the performance of PLS strategy for analyzing fused data. Finally, spectroscopic methods, combined with the SO-PLS-LDA method, offer a potential tool for classifying different types of milk samples and can be applied in various adulteration and authentication studies.

## Data availability

Data will be made available on request.

## CRediT authorship contribution statement

**Saeedeh Mohammadi:** Writing – review & editing, Writing – original draft, Visualization, Validation, Software, Methodology, Investigation, Formal analysis, Data curation, Conceptualization. **Aoife Gowen:** Writing – review & editing, Supervision, Software, Funding acquisition, Conceptualization. **Colm O'Donnell:** Writing – review & editing, Supervision.

## Declaration of competing interest

The authors declare that they have no known competing financial interests or personal relationships that could have appeared to influence the work reported in this paper.
