# Supplementary material for: Vibrational spectroscopy data fusion for enhanced classification of different milk types
Source: Heliyon. 2024 Aug 15;10(16):e36385. doi: 10.1016/j.heliyon.2024.e36385 (PMC11378925; doi:10.1016/j.heliyon.2024.e36385)
Supplement: Multimedia component 1 [file mmc1.docx]

**Vibrational Spectroscopy Data Fusion for Enhanced Classification of Different Milk Types**

Saeedeh Mohammadi, Aoife Gowen*, Colm O’Donnell*

Saeedeh.Mohammadi@ucdconnect.ie

*Aoife.Gowen@ucd.ie

[*Colm.Odonnell@ucd.ie](mailto:*Colm.Odonnell@ucd.ie)

School of Biosystems and Food Engineering, University College Dublin, Belfield, Dublin 4, Ireland

Table S1. Nutrition Information (Average Values per 100 ml) for different types of milk studied in this research

| **Milk Type**  **Parameter** | **Butter** | **Fresh** | **Heart Active** | **Lactose Free** | **Light** | **Protein** | **Slimline** |
| --- | --- | --- | --- | --- | --- | --- | --- |
| **Energy** | 254 kJ/61kcal | 267 kJ/64kcal | 188 kJ/45kcal | 171 kJ/41kcal | 178 kJ/42kcal | 211 kJ/50kcal | 160 kJ/38kcal |
| **Fat** | **3.0 g** | 3.5 g | **1.0 g** | 1.5 g | **1.0 g** | 1.0 g | **0.2 g** |
| **Of which saturates** | 1.6 g | 2.2 g | 0.6 g | 0.9 g | 0.6 g | 0.6 g | 0.1 g |
| **Carbohydrate** | 5.0 g | 4.7 g | 5.3 g | **3.4 g** | 4.8 g | 4.8 g | 5.2 g |
| **Of which sugars** | 5.0 g | 4.7 g | 4.8 g | **3.4 g** | 4.8 g | 4.8 g | 5.2 g |
| **Protein** | 3.4 g | 3.4 g | 3.5 | 3.4 g | 3.5 g | **5.4 g** | 3.8 g |
| **Salt** | 0.1 g | 0.11 g | 0.12g | 0.12 g | 0.11 g | 0.11 g | 0.12 g |
| **Calcium** | 120 mg | 119 mg | 122 mg | 118 mg | 123 mg | 172 mg | 123 mg |
| **Vitamin D3** | - | - | 1.0ug | 1.0ug | - | - | - |
| **Vitamin B12** | - | 0.4ug | 0.4ug | 0.46 ug | 0.4ug | 0.4ug | 0.4ug |

Table S2. Raw NIR, FT-IR, and Raman spectra for butter, fresh, heart active, lactose-free, light, protein, and slim line milk. Each column of the table shows a specific spectral modality, and each row from 1 to 7 shows different types of milk.

|  | NIR | FT-IR | Raman |
| --- | --- | --- | --- |
| 1 | 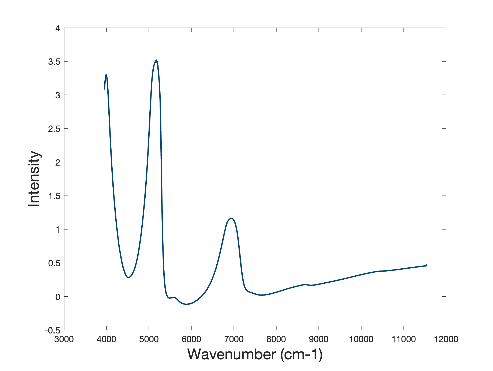 | 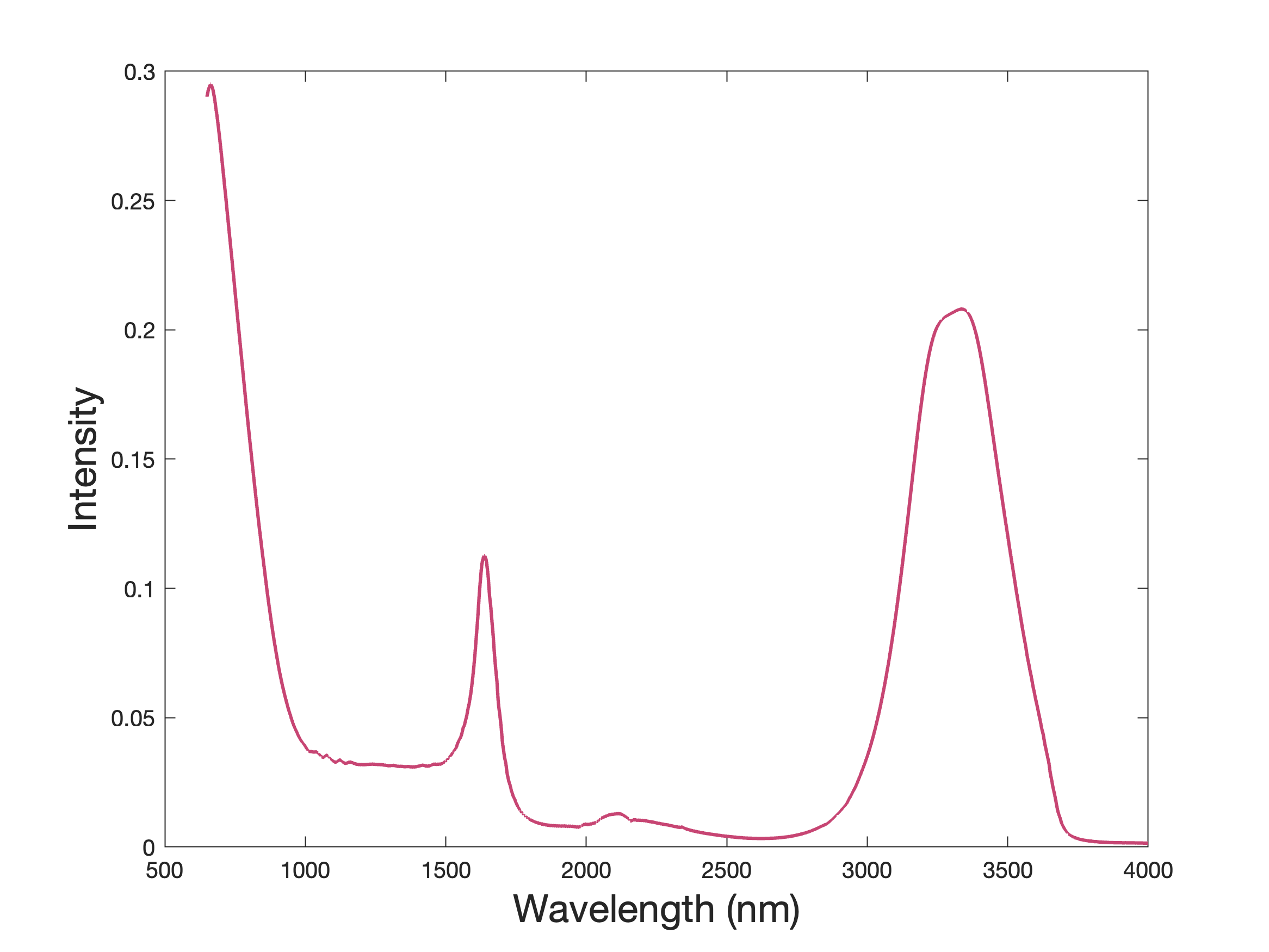 | 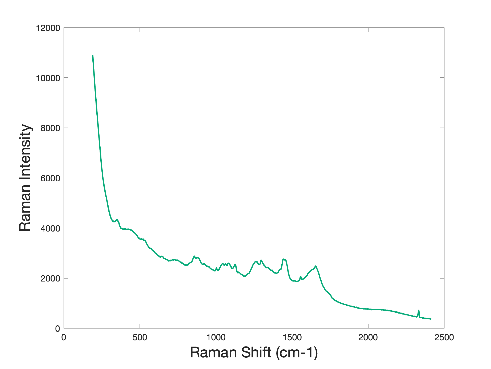 |
| 2 | 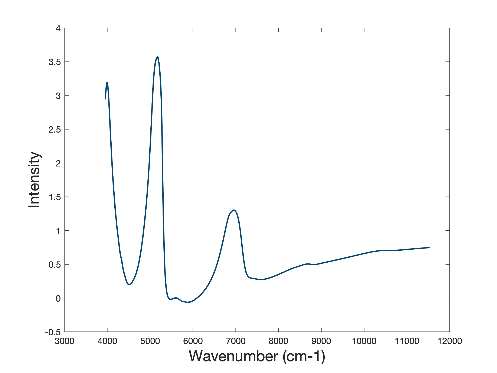 | 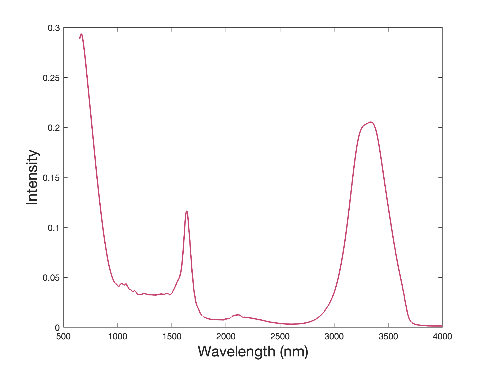 | 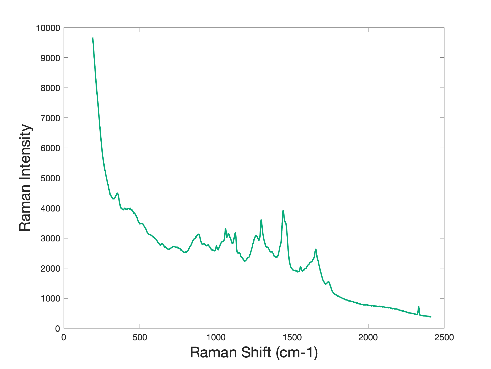 |
| 3 | 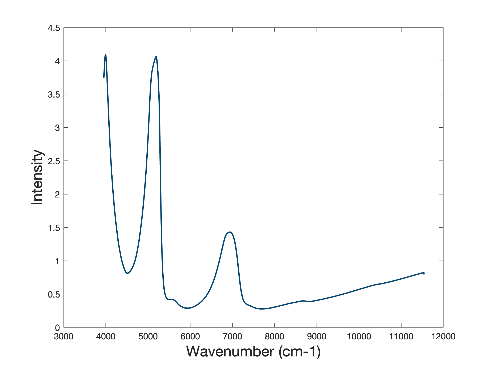 | 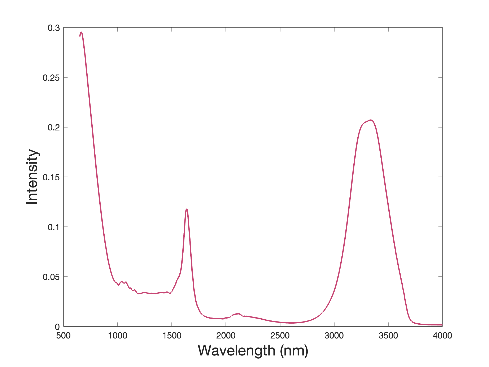 | 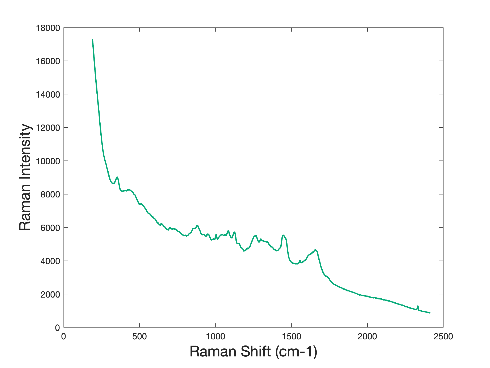 |
| 4 | 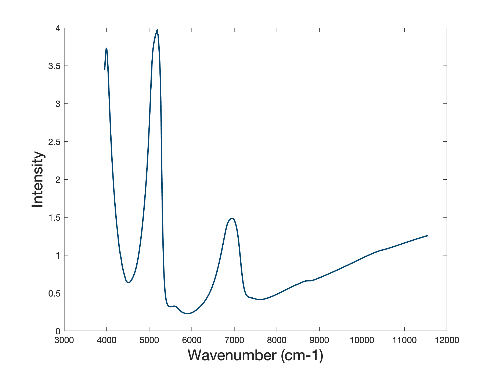 | 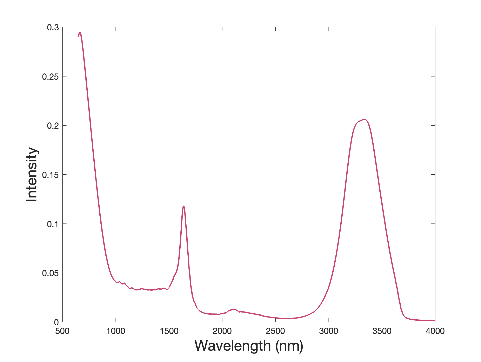 | 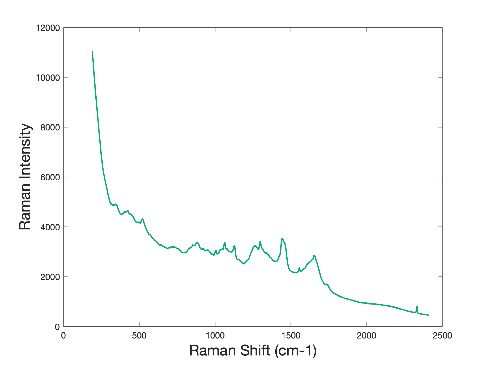 |
| 5 | 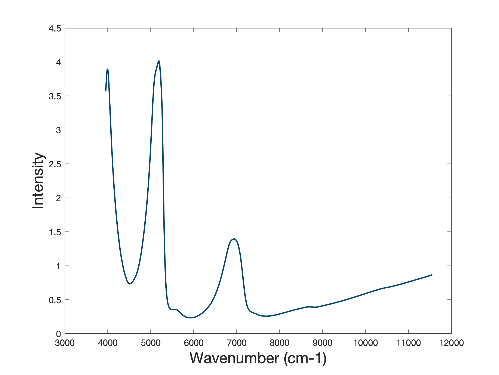 | 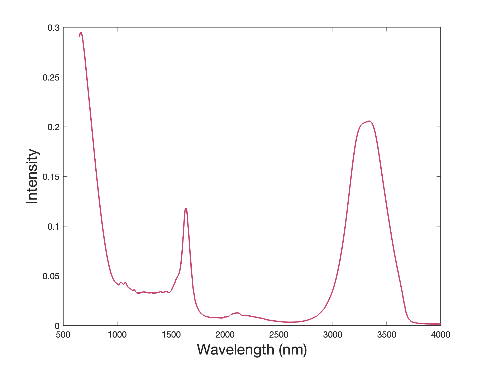 | 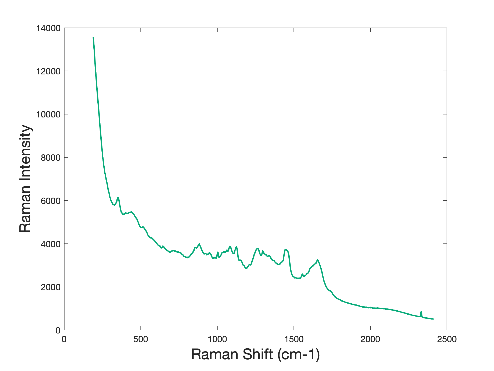 |
| 6 | 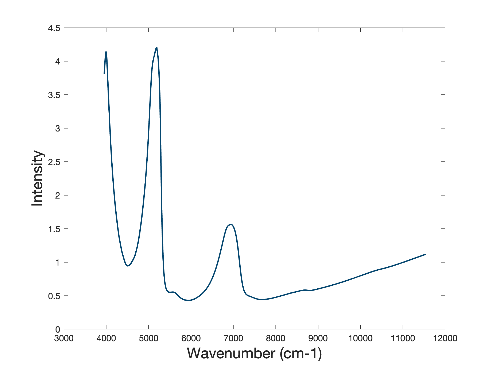 | 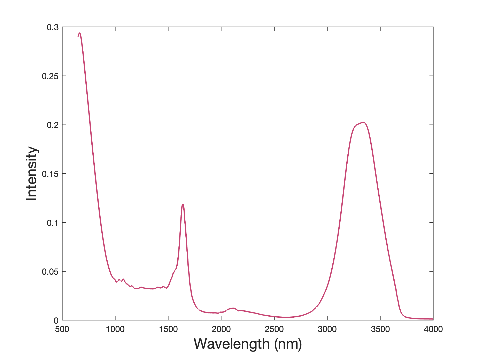 | 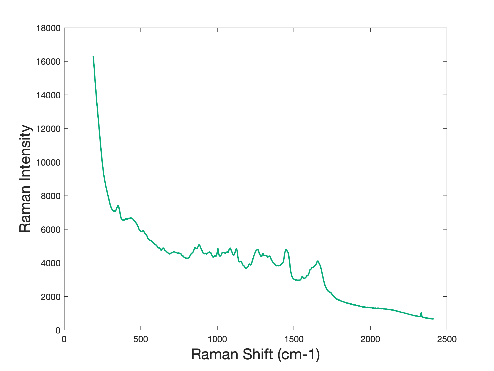 |
| 7 | 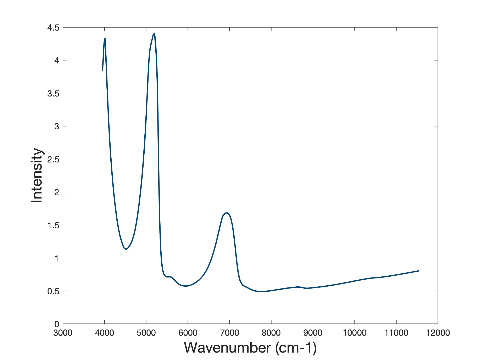 | 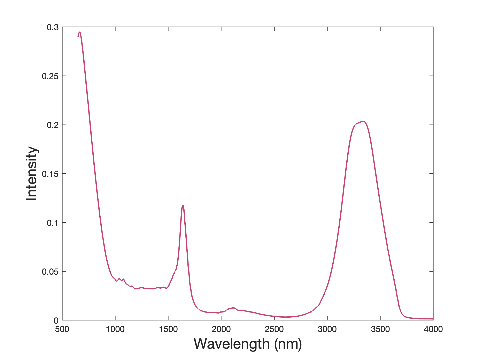 | 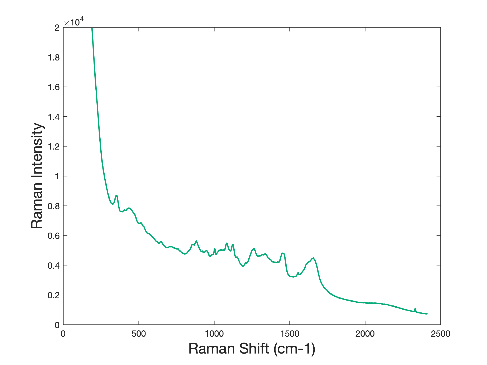 |


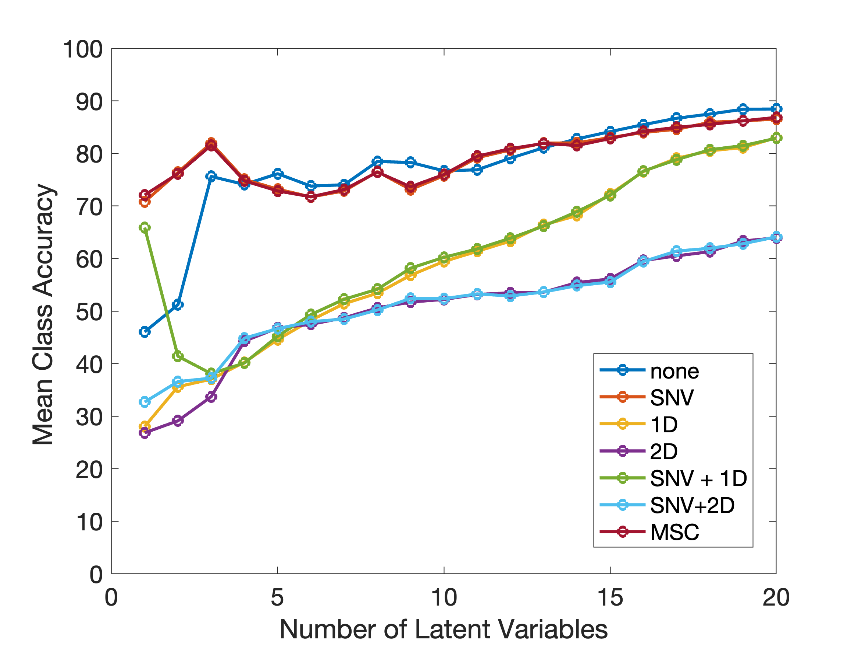


Figure S1. Mean class accuracy versus number of latent variables acquired from PLS-DA analysis of NIR data with cross validation. Each plot corresponds to one of the following: no pretreatment, SNV, first derivative, second derivative, first derivative after SNV, second derivative after SNV, and MSC


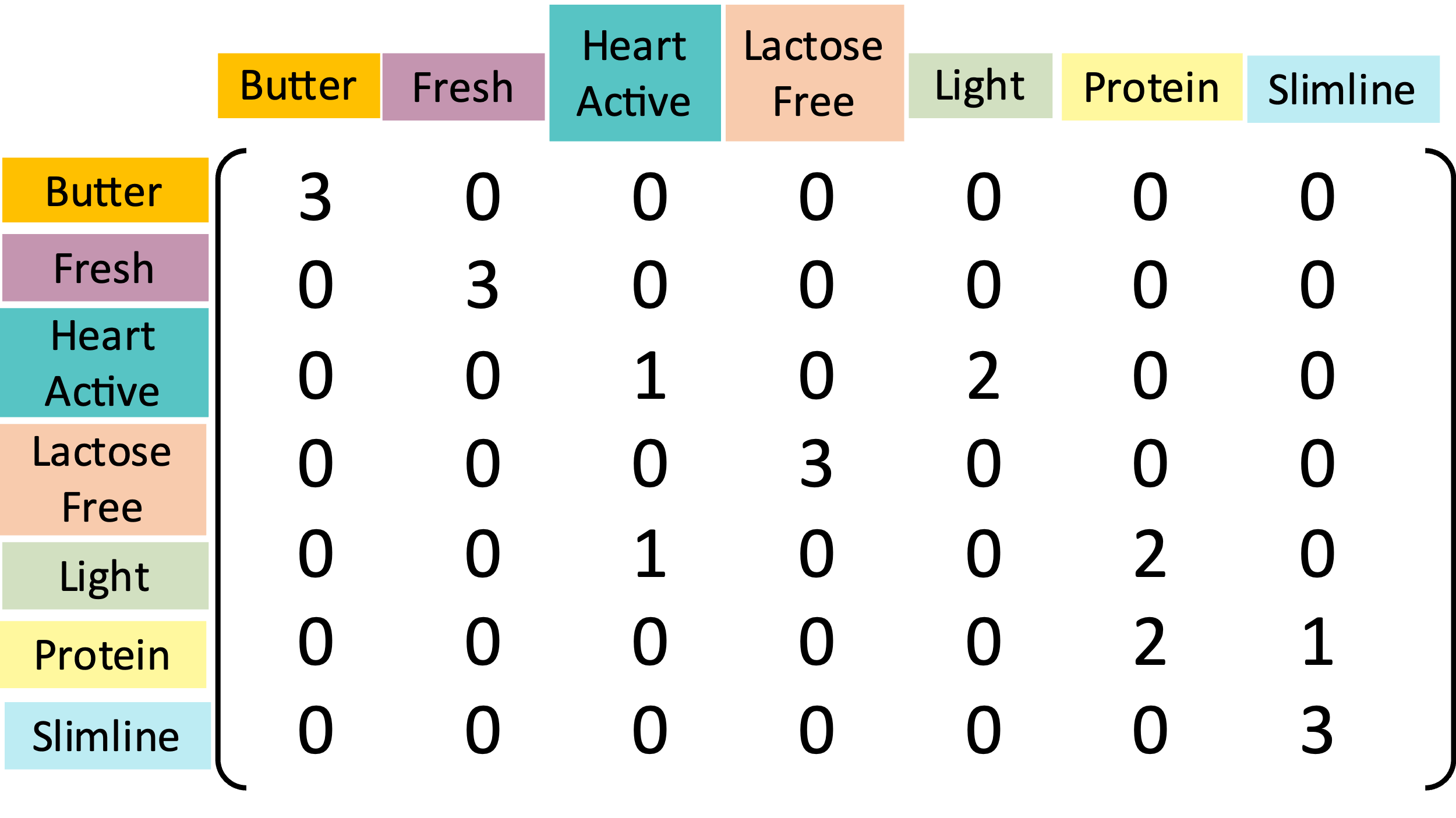


Figure S2. the confusion matrix of test set for PLS-DA analysis of NIR data


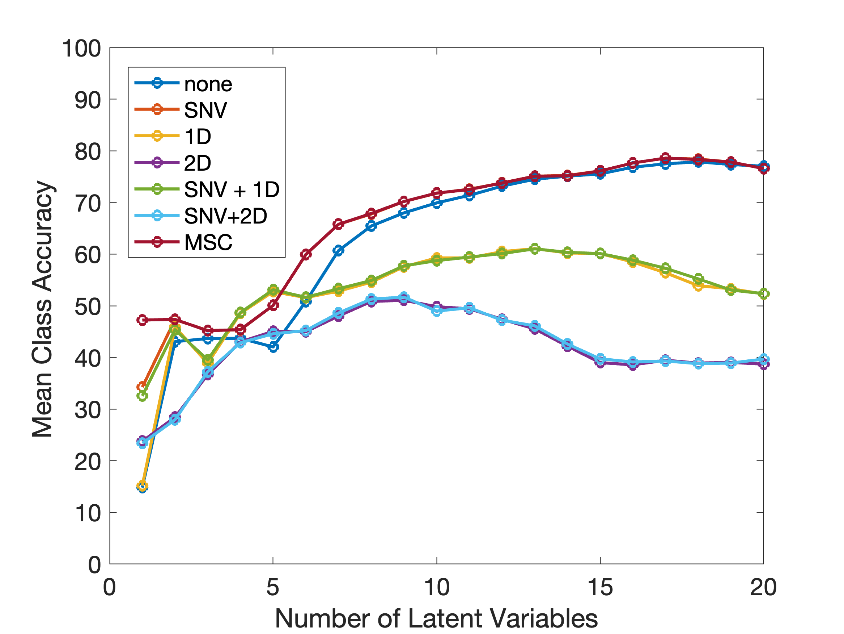


Figure S3. Mean class accuracy versus number of latent variables acquired from PLS-DA analysis of FT-IR data with cross validation. Each plot corresponds to one of the following: no pretreatment, SNV, first derivative, second derivative, first derivative after SNV, second derivative after SNV, and MSC


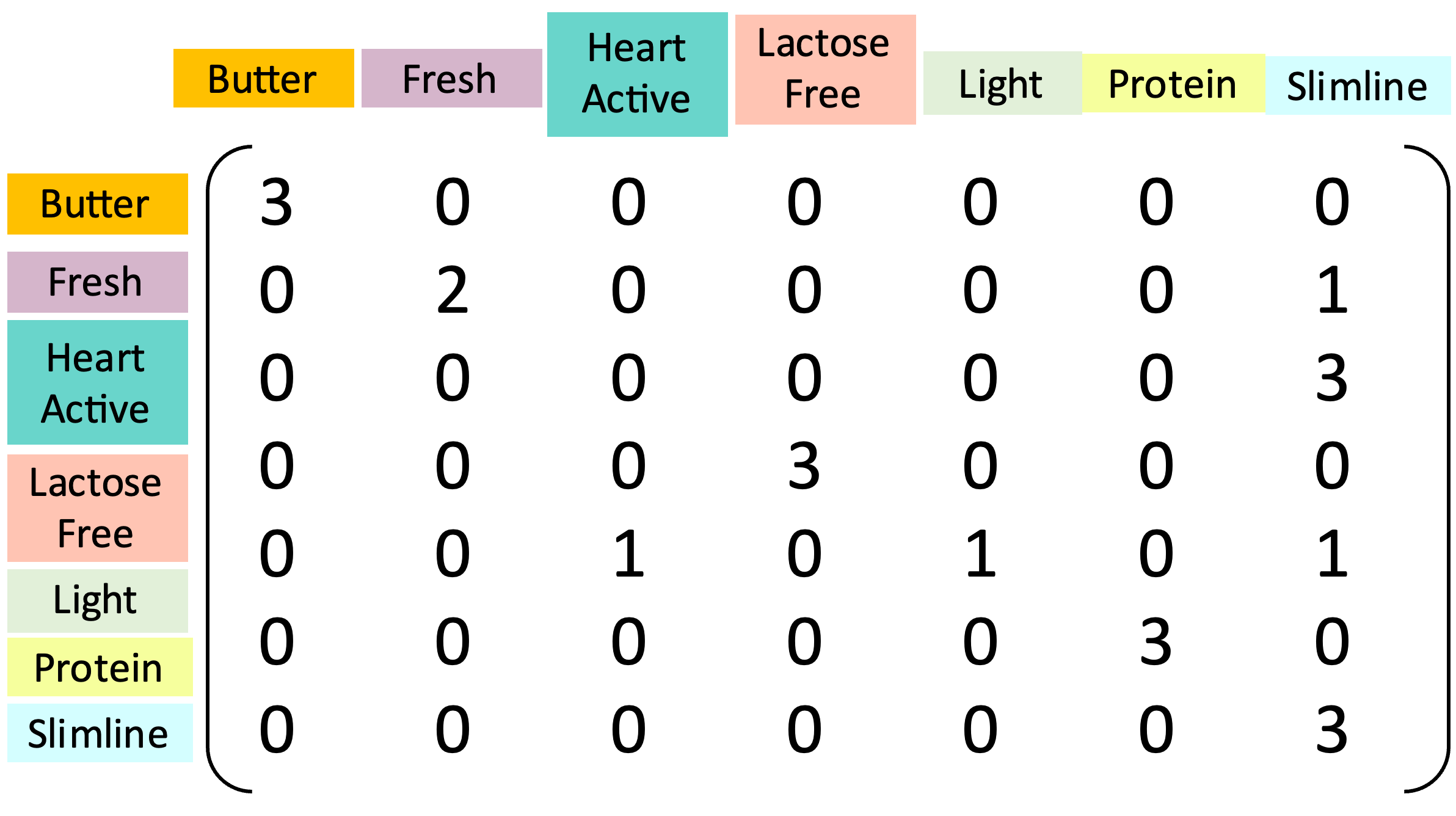


Figure S4. The confusion matrix of test set for PLS-DA analysis of FT-IR

**
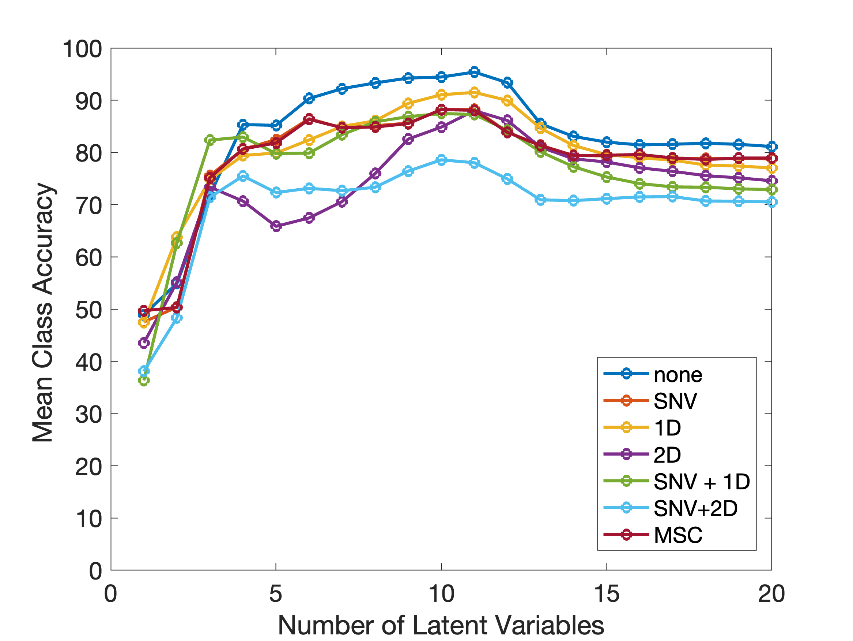
**

Figure S5. Mean class accuracy versus number of latent variables acquired from PLS-DA analysis of Raman data with cross validation. Each plot corresponds to one of the following: no pretreatment, SNV, first derivative, second derivative, first derivative after SNV, second derivative after SNV, and MSC

**
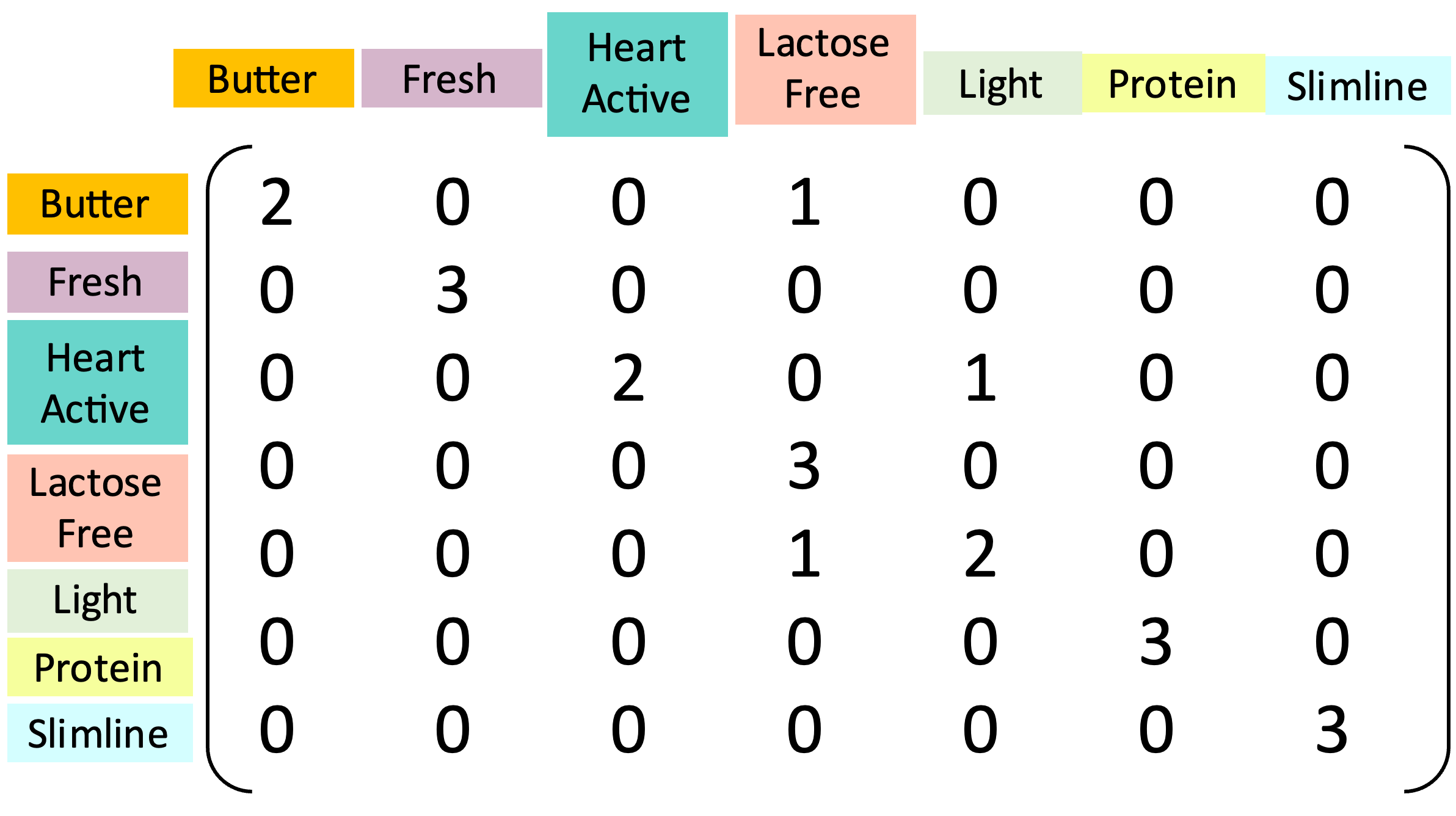
**

Figure S6. The confusion matrix of test set for PLS-DA analysis of Raman

Table S3. The accuracy of the prediction for different combination of pretreatment for three blocks of data

| **NO** | **Pretreatment for NIR** | **Pretreatment for FT-IR** | **Pretreatment for Raman** | **Accuracy of Prediction with Third Measurement as Validation Set** |
| --- | --- | --- | --- | --- |
| 1 | none | none | none | 0.86 |
| 2 | none | none | mean | 0.86 |
| 3 | none | none | auto | 0.90 |
| 4 | none | mean | none | 0.90 |
| 5 | none | mean | mean | 0.90 |
| 6 | none | mean | auto | 0.90 |
| 7 | none | auto | none | 0.95 |
| 8 | none | auto | mean | 0.95 |
| 9 | none | auto | auto | 0.90 |
| 10 | mean | none | none | 0.90 |
| 11 | mean | none | mean | 0.90 |
| 12 | mean | none | auto | 0.90 |
| 13 | mean | mean | none | 0.95 |
| **14** | mean | mean | mean | 0.90 |
| 15 | mean | mean | auto | 0.90 |
| 16 | mean | auto | none | 0.86 |
| 17 | mean | auto | mean | 0.90 |
| 18 | mean | auto | auto | 0.90 |
| 19 | auto | none | none | 0.90 |
| **20** | auto | none | mean | 0.90 |
| **21** | auto | none | auto | 0.86 |
| 22 | auto | mean | none | 0.90 |
| 23 | auto | mean | mean | 0.90 |
| 24 | auto | mean | auto | 0.86 |
| 25 | auto | auto | none | 0.90 |
| 26 | auto | auto | mean | 0.90 |
| 27 | auto | auto | auto | 0.86 |
